# RELIGIOUS EXPERIENCE AS A KEY FACTOR IN THE DEVELOPMENT OF LATE-LIFE TRUE BELIEF

**DOI:** 10.1093/geroni/igz038.1934

**Published:** 2019-11-08

**Authors:** Austin Prusak, Margaret Iapalucci, Eoin Walsh, Ann-Kathrin Wolfram, Andrew Futterman

**Affiliations:** 1 Loyola University Maryland, Baltimore, Maryland, United States; 2 Department of Psychology, Loyola University Maryland, Baltimore, Maryland, United States

## Abstract

Hoffer (1951) argued that “true believers” are individuals who cannot tolerate questions about their views. In terms of “true” religious believers, such individuals would not be able to tolerate questions about religious doctrine. In this study, we examine potential causes of religious “true” belief in later life. Using a model of religious orientation developed by Allport (1950) and Batson (1991), we defined “true” religious believers as those individuals who describe deep and broad religious commitments (score high on measures of intrinsic religious orientation) but refuse to question or doubt their commitments (score low on quest religious orientations). Using logistic regression analyses, we examined predictors of religious true belief in a sample of 357 denominationally diverse older adults living in New England. Predictors in the model included denominational affiliation, religious knowledge, organizational and non-organizational religious participation, and religious experiences, as well as non-religious factors such as personality traits (NEO), severity of illness and self-rated health, overall life stress, and other background factors (SES, gender, age) Of these 14 predictors, one factor in particular stood out as a particularly robust predictor of membership in the “true” believer group (n=47) – high scores on self-reported religious experience. Religious life experiences may be influenced by cohort and personality, resulting in extremely high scores of the individual on the intrinsic orientation scale and extremely low scores on the quest scale; together these embody the “true believer”.

